# Burden of disease and impact on quality of life in chronic back pain – a comparative cross-sectional study of 150 axial spondyloarthritis and 150 orthopedic back pain patients

**DOI:** 10.3389/fmed.2023.1221087

**Published:** 2023-08-17

**Authors:** Natalie Frede, Sonja Hiestand, Dominique Endres, Ludger Tebartz van Elst, Stephanie Finzel, Nina Chevalier, Markus A. Schramm, Ina C. Rump, Jens Thiel, Reinhard Voll, Georg Herget, Nils Venhoff

**Affiliations:** ^1^Department of Rheumatology and Clinical Immunology, Medical Center - University of Freiburg, Faculty of Medicine, University of Freiburg, Freiburg, Germany; ^2^Department of Psychiatry and Psychotherapy, Medical Center - University of Freiburg, Faculty of Medicine, University of Freiburg, Freiburg, Germany; ^3^Division of Rheumatology and Clinical Immunology, Medical University Graz, Graz, Austria; ^4^Department of Orthopedics and Trauma Surgery, Medical Center - University of Freiburg, Faculty of Medicine, University of Freiburg, Freiburg, Germany

**Keywords:** chronic back pain, mechanical back pain, axial spondyloarthritis, quality of life, depression, functional impairment

## Abstract

**Objective:**

Chronic back pain (CBP) constitutes one of the most common complaints in primary care and a leading cause of disability worldwide. CBP may be of mechanical or inflammatory character and may lead to functional impairment and reduced quality of life. In this study, we aimed to assess and compare burden of disease, functional capacity, quality of life and depressive symptoms in axial spondyloarthritis (axSpA) patients with orthopedic chronic back pain patients (OBP). We further aimed to identify factors associated with quality of life.

**Methods:**

Cross-sectional survey of a cohort of 300 CBP patients including 150 patients from a University Hospital Orthopedic Back Pain Outpatient Clinic with OBP and 150 patients with confirmed axSpA from a University Hospital Rheumatology Outpatient Clinic. Questionnaire-based assessment of pain character (Inflammatory Back Pain, MAIL-Scale), functional status (FFbH, BASFI), quality of life (WHOQOL-Bref) and depressive symptoms (Phq9) and retrospective medical chart analysis.

**Results:**

Both, OBP and axSpA patients reported on average intermediate pain levels of mostly mixed pain character. Both groups demonstrated a reduced health-related quality of life and the presence of depressive symptoms. However, axSpA patients reported a significantly better subjective quality of life, more satisfaction with their health status and better functional capacity compared to OBP patients (all *p* < 0.001). In a multivariate regression model, depressive symptoms, mechanical back pain, pain level and age were negative predictors of subjective quality of life, whereas functional capacity was a positive predictor.

**Conclusion:**

Chronic back pain was associated with a high morbidity and reduced quality of life regardless of pain character. We identified multiple factors associated with reduced quality of life. Awareness and addressing of these factors may help to overcome unmet needs and improve quality of life for these patients.

## 1. Introduction

Back pain constitutes one of the most common complaints in primary care (1). Lifetime prevalence has been estimated to be 70–85% and up to 50% of working adults experience back pain every year (2–4). While most episodes are self-limited with recovery or significant improvement in 90% of cases within 3 months, chronic back pain constitutes a common health issue and a major cause of disability and work absence (5).

Regarding pain character, back pain can be broadly categorized as mechanical, inflammatory or referred back pain. Mechanical back pain is responsible for approximately 97% of chronic back pain cases, while non-mechanical causes include rheumatic diseases, vascular or malignant causes or infections (6). Mechanical back pain is commonly characterized by intermittent pain during the day or pain that develops later in the day, pain during certain movements or upon mechanical strain such as lifting, trunk flexion or extension or standing for a while (7). Common causes of mechanical back pain include degenerative disc disease, spinal stenosis, vertebral fractures, facet or sacroiliac osteoarthritis and myogenic or myofascial pain (6). In contrast, inflammatory back pain is characterized by morning stiffness, nocturnal pain with early awakening and improvement upon exercise (8). Inflammatory back pain is a clinical hallmark of axial spondyloarthritis (axSpA). With a prevalence of 0.5%, axSpA constitutes one of the most common inflammatory rheumatic diseases in Europe (9). While a predominantly axial manifestation with spondylitis and/or sacroiliitis presenting as inflammatory back pain is typical, axSpA may also be associated with peripheral arthritis, enthesitis/tendinitis as well as uveitis, which may contribute to an increased morbidity (10, 11). A diagnostic latency of multiple years is still common due to an often insidious onset of symptoms and lack of awareness (12). However, delayed or inadequate treatment of the disease can lead to irreversible spinal changes and long-term functional impairment. It has been previously shown that women less commonly demonstrate the typical inflammatory back pain as a presenting symptom and thus diagnostic delay and misdiagnosis are more common in female patients (13). Sex differences in axSpA presentation, symptoms and therapy response have recently gained increased interest (14).

Chronic back pain may have a major impact on health-related quality of life (QoL) (15). Among other factors, pain severity and disability contribute to a reduced QoL (16). However, also the psychological status was identified as a significant contributor (17). Chronic pain and specifically also chronic back pain have been associated with impaired mental health and an increased occurrence of depression (18, 19).

Within this study, we aimed to assess the pain character, burden of disease, functional status, QoL, symptoms of depression and sex differences in a cohort of 300 back pain patients including 150 patients with orthopedic back pain (OBP) causes and 150 patients with axSpA and identify factors associated with QoL.

## 2. Materials and methods

### 2.1. Patient cohort

For this cross-sectional study, a total of 300 chronic back pain patients were recruited, including 150 patients from the from the Orthopedics outpatient clinic and 150 patients from the Rheumatology outpatient clinic of the University Medical Center Freiburg, Freiburg, Germany. Consecutive patients presenting to the Rheumatology outpatient clinic with axSpA, respectively all patients presenting to the Orthopedic Outpatient clinic due to chronic back pain were asked to participate in the study. AxSpA patients had to fulfil the modified New York (mNY) or Assessment of Spondyloarthritis International Society (ASAS) criteria. OBP patients needed to suffer from back pain for at least 6 weeks. Specific diagnoses of OBP patients are listed in section 3.1. This study was conducted under the ethics protocol 37/17 of the ethics committee of the University of Freiburg, Germany. Patients gave their consent according to International Conference on Harmonization Good Clinical Practice (ICH GCP) guidelines. The results section of this manuscript comprises a detailed description of the patient cohort.

### 2.2. Instruments/measures

#### 2.2.1. Back pain

Pain intensity was assessed on a visual analogue scale of 0-100 mm, representing a continuum between “no pain” and “worst imaginable pain.”

To screen for symptoms of inflammatory back pain, the Inflammatory Back Pain questionnaire consisting of 5 items was used (10). The questionnaire consists of dichotomous questions on age of onset, pain localization to buttocks, nightly awakening, improvement with exercise and improvement with non-steroidal anti-inflammatory drugs (NSAIDs). A score of ≥3 points on this scale is suspicious of axSpA with a sensitivity of 79% and a specificity of 49%.

To further characterize lower back pain, the Mechanical and Inflammatory Low Back Pain Scale (MAIL-Scale) was employed (7). The MAIL Scale consists of 19 questions relating to notional inflammatory (Part A) and mechanical (Part B) back pain asking the patient to answer ‘yes’ or ‘no’. Part A consists of 6 signs and symptoms associated with inflammatory back pain and gives a maximum score of 16 points, whereas Part B consists of 13 signs and symptoms associated with mechanical back.

pain and gives a maximum score of 13 points. Scoring was conducted according to the instructions provided by Riksman et al. Mean scores of Part A and Part B were used as cut-offs. Patients with greater than mean scores in one and less than mean in the other subscale were classified as purely inflammatory or purely mechanical back pain. All other patients were classified as having “mixed” back pain.

Disease activity was furthermore measured with Bath Ankylosing Spondylitis Disease Activity Index (BASDAI) (20), which constitutes a validated instrument to assess disease activity in axial spondyloarthritis. The BASDAI consists of six items assessing fatigue, spinal pain, arthralgia, enthesial complaints and morning stiffness rated on a visual analogue scale of 0–10. Scores of ≥4 indicate suboptimal disease control.

#### 2.2.2. Functional capacity

Functional capacity was assessed with the help of Bath Ankylosing Spondylitis Functional Index (BASFI), which constitutes a validated instrument for the assessment of functional status and impairment in axSpA patients (21). The BASFI consists of 10 items assessing activities of everyday life, which are rated on a scale of 0 (no impairment) to 10 (severe impairment). A study by Maksymowych et al. reported a score of < 3 to constitute an acceptable symptom state (22).

Furthermore, functional capacity in everyday life was additionally assessed employing the Functional Questionnaire Hannover (Funktionsfragebogen Hannover, FFbH), which constitutes a tool developed in Germany to assess functional capacity in patients with rheumatic diseases or other joint problems (23). The FFbH comprises 18 items and assesses whether the patients are able to complete 18 different tasks of everyday life either independently without difficulties (2 points), with effort (1 point) or unable/need help (0 points). Scored points are multiplied by 100 and divided by 2x the number of valid answers. On this scale, relevant functional impairment is defined as a functional capacity <60%.

#### 2.2.3. Quality of life

To assess QoL, the WHO-QOL-Bref questionnaire was used (short version of the World Health Organisation Quality of Life questionnaire), which constitutes a validated and well-established instrument for the assessment of subjective QoL (24). The questionnaire has 26 items assessing well-being within the previous 2 weeks, rated on a five-point Likert scale from very poor/very unsatisfactory to very good/very satisfactory. The scale is divided into the subdomains of subjective QoL, physical and mental health-related QoL, social relationships and environmental QoL. Domain scores are calculated from the average score of all items within the domain and transformed into a 0–100 scale. Higher scores indicate a better QoL.

#### 2.2.4. Depression

Depressive symptoms were assessed with the Patient Health Questionnaire 9 (Phq9), which comprises 9 items to screen for depression (25). Symptoms occurring within the last 2 weeks are scored on a Likert scale of 0 (not at all) to 3 (nearly every day). Assessed symptoms include anhedonia, low mood, sleep, fatigue, appetite, guilt, concentration, motor disturbance and suicidal thoughts. Total scores range from 0 and 27, with cutoff points of 5, 10, 15 and 20 indicating mild, moderate, moderately severe and severe symptoms of depression.

### 2.3. Demographic variables

In addition, retrospective medical chart analysis was performed to confirm the diagnosis, collect data on current or recommended therapies as well as disease duration for OBP and axSpA patients. Furthermore, demographic data on sex, age, height, body weight, body mass index (BMI), smoking status as well as highest educational degree (no degree, lower secondary education, intermediate school certificate, polytechnic entrance qualification, high school diploma, polytechnic degree, university degree, postgraduate) were collected.

### 2.4. Statistical analysis

Descriptive statistics such as percentages, mean and standard variation were employed to depict demographic variables and disease characteristics. Chi-square test was used to compare categorical variables. For continuous variables, Student’s *t*-test was used for normally distributed variables and Welch’s t-test for variables with unequal variance.

Multivariate linear regression was employed to predict factors independently associated with subjective QoL. Models were constructed by stepwise backwards elimination. The statistical level of significance was set at *p* < 0.05.

Statistical analyses were performed using GraphPad Prism software version 9.5.1 for Mac, GraphPad Software, San Diego, California United States”^1^ or Jamovi version 2.3.21.0. (The jamovi project (2021). *Jamovi*. (Version 2.3) [Computer Software], retrieved from)^2^ (26).

## 3. Results

### 3.1. Patient cohort

A total of 300 patients with back pain were surveyed, including 150 patients consulting the orthopedic outpatient clinic due to back pain and 150 patients with confirmed axSpA from the rheumatology outpatient clinic. In the OBP cohort (*n* = 150) patients had degenerative disc disease/spinal disc herniation (*n* = 39), sacroiliac joint syndrome (*n* = 19), vertebral fractures (*n* = 17), spinal stenosis (*n* = 16), facet syndrome/osteoarthritis (*n* = 15), cervical syndrome/thoracic spine syndrome/lumbar syndrome (*n* = 14), postoperative complaints after spinal surgery (*n* = 13), spondylolisthesis (*n* = 7), scoliosis (*n* = 5) and spondylitis/spondylodiscitis (*n* = 5).

In the axSpA cohort (*n* = 150), axial involvement was present in 100% of patients, peripheral arthritis in 42%, enthesitis in 26% and dactylitis in 6.7% of patients. History of uveitis/eye involvement occurred in 20.7%, psoriasis in 19.5% and inflammatory bowel disease in 5.3% of patients. HLA B27 positivity was confirmed in 74.3% of patients and 29.5% had a positive family history. Mean disease duration was 10.9 years (min. 0 years, max. 58 years).

Out of the 300 surveyed patients, 57.3% were male (OBP 55.3%, axSpA 59.3%) and 42.7% female (OBP 44.7%, axSpA 40.7%). Mean age of axSpA patients was 48.99 years (min. 21, max. 90 years) compared to 56.74 years (min. 22, max. 86 years) in the OBP group (*p* < 0.001, see Table 1). Smoking was reported by 28.1% of patients; there were no significant differences regarding smoking status between the axSpA and OBP subgroups. The average BMI of both, the OBP and the axSpA patients, was within the overweight spectrum (OBP 27.25 kg/m^2^ vs. axSpA 26.83 kg/m^2^, *p* = 0.575).

**Table 1:** Patients characteristics.

		Total (*n* = 300)	axSpA (*n* = 150)	OBP (*n* = 150)	value of *p*
Age, years, mean (SD)		52.9 (16.0)	48.99 (15.6)	56.74 (15.4)	**<0.001**
Male, *n* (%)/Female, *n* (%)		172 (57.3%)/128 (42.7%)	89 (59.3%)/61 (40.7%)	83 (55.3%)/67 (44.7%)	0.484
BMI, kg/m^2^, mean (SD) (*n* = 257)		27.0 (5.83)	26.8 (5.73)	27.2 (5.99)	0.575
Smokers, *n* (%) (*n* = 242)		68 (28.1%)	39 (29.1%)	29 (26.9%)	0.698
Disease duration, years, mean (SD)		n.a.	10.9 (11.0)	n.a.	n.a.
Highest educational degree (*n* = 282)	Postgraduate, *n* (%)	6 (2.1)	6 (4.2)	0 (0.0)	**0.018**
	University degree, *n* (%)	26 (9.2)	17 (11.8)	9 (6.5)	
	Polytechnic degree, *n* (%)	29 (10.3)	12 (8.3)	17 (12.3)	
	High school diploma (Abitur), *n* (%)	19 (6.7)	9 (6.3)	10 (7.2)	
	Polytechnic entrance qualification, *n* (%)	23 (8.7)	11 (7.6)	12 (8.7)	
	Intermediate school certificate, *n* (%)	86 (30.5)	52 (36.1)	34 (24.6)	
	Lower secondary education, *n* (%)	83 (29.4)	33 (32.9)	50 (36.2)	
	No degree, *n* (%)	10 (3.5)	4 (2.8)	6 (4.3)	
Therapy	bDMARD, *n* (%)	–	92 (62.6)	–	n.a.
	csDMARD, *n* (%)	–	27 (18.4)	–	n.a.
	NSAID monotherapy, *n* (%)	–	22 (15.0)	–	n.a.
	No therapy, *n* (%)	–	19 (12.9)	–	n.a.
	Surgery, *n* (%)	–	–	34 (22.7)	n.a.
	Injection therapy, *n* (%)	–	–	24 (16.0)	n.a.
	Physical therapy/ analgesics, *n* (%)	–	–	78 (52.0)	n.a.
	Orthopedic aids, *n* (%)	–	–	8 (5.3)	n.a.
	Further investigations/no therapy, *n* (%)	–	–	6 (4.0)	n.a.

Bold values are statistically significant with a *p* < 0.05. For parameters for which data from individual patients were missing, the *n* number for the individual item is given on the left. axSpA, axial spondyloarthritis; OBP, orthopedic back pain; SD, standard deviation; *n*, number; BMI, body mass index; csDMARD, conventional synthetic disease modifying antirheumatic drug; bDMARD, biological DMARD; NSAID, non-steroidal anti-inflammatory drugs; n.a., not available.

Regarding the highest educational degree, 59.9% of the patients interviewed had a lower or intermediate secondary school-leaving qualification (OBP 60.8%, axSpA 69.0%). A polytechnic entrance qualification or high school diploma were reported as the highest educational degree by 15.4% of the patients (OBP 15.9%, axSpA 13.9%). In total, 21.6% had a polytechnic or university degree or were postgraduated (OBP 18.8%, axSpA 24.3%) whereas 3.5% of the patients had no school-leaving qualification (OBP 4.3%, axSpA 2.8%). Overall, the SpA patients thus possessed a slightly higher level of education than the OBP patients (*p* = 0.018).

At the timepoint of survey, 62.6% of axSpA patients received a biological disease modifying antirheumatic drug (bDMARD), 18.4% a conventional synthetic (cs)DMARD and 15.0% NSAIDs while 12.9% did not receive any therapy. In the OBP group, 22.7% underwent surgery for their back problems and 16% received an injection therapy of facet or SI joints. Physiotherapy and analgesics were recommended in 52 and 5.3% received orthopedic aids (brace, orthosis, etc.). Further investigations or no specific therapy were recommended in 4% of patients.

### 3.2. Characterization of back pain

Regarding their current pain intensity, both the OBP and the axSpA cohorts reported on average moderate pain levels. Pain intensity was slightly higher in the OBP group, marginally missing level of significance (VAS 49.53/100 vs. 42.16/100, *p* = 0.051, see Table 2). AxSpA patients significantly more often reported an onset of back pain before the 35th year of life (*p* < 0.001). Morning stiffness did not differ significantly between both groups (OBP 66.7% vs. axSpA 73.3%, *p* = 0.216). Awakening due to back pain was frequent in both subgroups (OBP 47.1% vs. axSpA 53.8%, *p* = 0.260). Improvement with exercise was significantly more common in the axSpA cohort (88.3% vs. 58.3%, *p* < 0.001), whereas the OBP patients significantly more often reported continuous pain (54.5% vs. 36.4%, *p* = 0.002). In the cohorts of OBP and axSpA 75.0 and 80.3% of patients reported an improvement on NSAID treatment (*p* = 0.312), respectively.

**Table 2:** Characteristics of back pain.

	Total (*n* = 300)	axSpA (*n* = 150)	OBP (*n* = 150)	value of *p*
Pain intensity (VAS 0–100), mean (SD)	45.4 (29.8)	42.16 (26.87)	49.53 (32.72)	0.051
Inflammatory Back Pain questionnaire, mean (SD)	2.83 (1.28)	3.23 (1.28)	2.42 (1.16)	**<0.001**
Morning stiffness, *n* (%) (*n* = 293)	205 (70.0)	107 (73.3)	98 (66.7)	0.216
Morning stiffness >30 min, *n* (%) (*n* = 291)	69 (23.7)	36 (24.3)	33 (23.1)	0.802
Start of symptoms <35 years of age, *n* (%) (*n* = 295)	172 (58.3)	108 (73.5)	64 (43.2)	**<0.001**
Awakening due to back pain, *n* (%) (*n* = 283)	143 (50.5)	78 (53.8)	65 (47.1)	0.260
Improvement with exercise, *n* (%) (*n* = 269)	198 (73.6)	121 (88.3)	77 (58.3)	**<0.001**
Improvement with NSAID treatment, *n* (%) (*n* = 252)	196 (77.8)	106 (80.3)	90 (75.0)	0.312
Continuous pain, *n* (%) (*n* = 286)	130 (45.5)	52 (36.4)	78 (54.5)	**0.002**
Pain upon standing for a while, *n* (%) (*n* = 285)	228 (80.0)	104 (72.2)	124 (87.9)	**<0.001**
Pain upon lifting, *n* (%) (*n* = 278)	169 (60.8)	63 (45.0)	106 (76.8)	**<0.001**
Pain upon bending forward, *n* (%) (*n* = 271)	157 (57.9)	67 (46.5)	90 (70.9)	**<0.001**
Bath Ankylosing Spondylitis Disease Activity Index (BASDAI), mean (SD)	3.96 (2.24)	3.66 (2.23)	4.37 (2.21)	**0.013**
MAIL Scale Inflammatory scale, mean (SD)	6.70 (4.14)	6.53 (4.09)	6.87 (4.19)	0.469
MAIL Scale Mechanical scale, mean (SD)	6.53 (3.72)	5.68 (3.91)	7.38 (3.33)	**<0.001**
Bath Ankylosing Spondylitis Functional Index (BASFI), mean (SD)	3.38 (2.45)	2.95 (2.41)	3.88 (2.41)	**0.002**
Functional Capacity (FFbH), mean (SD)	75.3 (20.2)	80.8 (18.4)	69.7 (20.5)	**<0.001**
Impaired functional capacity, *n* (%)	72 (25.4)	22 (15.4)	50 (35.7)	**<0.001**

Bold values are statistically significant with a *p* < 0.05. For parameters for which data from individual patients were missing, the *n* number for the individual item is given on the left. axSpA, axial spondyloarthritis; OBP, orthopedic back pain; SD, standard deviation; *n*, number; VAS, visual analogue scale.

On the Inflammatory Back Pain questionnaire, the OBP patients scored an average of 2.42 points compared to 3.23 points in the axSpA cohort (*p* < 0.001). However, 47.3% of OBP patients reached three or more points, suspicious of spondyloarthritis (sensitivity for SpA 79%, specificity 47%, i.e., diagnosis of SpA expected in every second patient). In the BASDAI, the OBP patients averaged 4.37 points, compared to 3.66 points in the axSpA patients (*p* = 0.013).

To further differentiate inflammatory and mechanical lower back pain, the MAIL-Scale questionnaire was used. In Part A, which assesses characteristics of inflammatory low back pain, OBP patients scored an average of 6.87 points whereas axSpA patients scored 6.53 out of a possible 16 points (*p* = 0.469). In Part B, which relates to mechanical back pain, the OBP cohort averaged 7.38 points out of a possible 13 points, compared to a mean of 5.68 points in the axSpA cohort (*p* < 0.001). According to the analysis after Riksman et al. (7), in both cohorts the majority of patients thus had back pain of mixed pain character with both inflammatory and mechanical pain components. However, mechanical back pain symptoms significantly correlated with age (*p* = 0.023).

Regarding functional capacity, the OBP cohort reached on average 69.7% in the FFbH, measuring functional capacity in everyday life, compared to 80.8% in the axSpA cohort (*p* < 0.001). 35.7% of the OBP and 15.4% of SpA patients had a clinically relevant functional impairment (<60% FFbH, *p* < 0.001). OBP patients scored an average of 3.88 in the BASFI compared to 2.95 points in the axSpA cohort (*p* = 0.002).

Comorbid fibromyalgia occurred in 11.4% of axSpA patients. Data on the frequency of fibromyalgia in orthopedic back pain patients were unfortunately not available.

### 3.3. Quality of life and depressive symptoms

**Figure 1:**
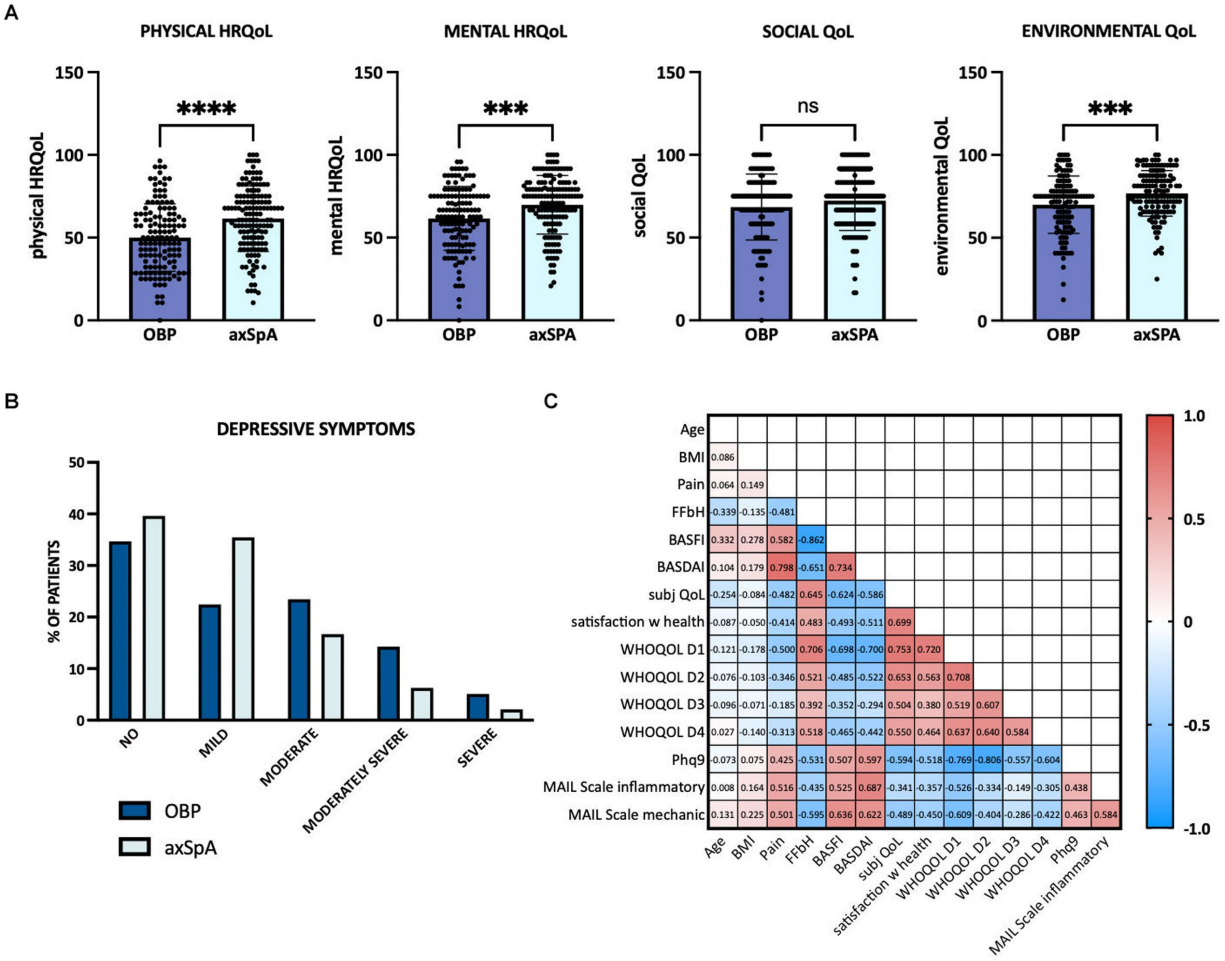
Quality of life, depressive symptoms and associated factors. **(A)** Physical health-related quality of life (HRQOL), mental HRQOL, social and environmental quality of life (WHOQOL, domains 1–4) depicted for chronic OBP patients in dark blue and axSpA patients in light blue. **(B)** Depressive symptoms determined by Phq9. OBP patients shown in dark blue, axSpA patients in light blue. **(C)** Pearson’s correlations between clinical data, quality of life and depressive symptoms. Numbers within the graph represent Pearson’s *R* values. Red colour indicates positive correlation, blue negative correlation. axSpA, axial spondyloarthritis; BASDAI, Bath Ankylosing Spondylitis Disease Activity Index; BASFI, Bath Ankylosing Spondylitis Functional Index; BMI, body mass index; FFbH, Funktionsfragebogen Hannover to measure functional capacity; MBP, mechanical back pain; WHOQOL D1, WHO Quality of Life Domain 1 (=physical HRQOL); D2, mental HRQOL; D3, social QOL; D4, environmental QOL; Phq9, patient health questionnaire-9 to screen for depressive symptoms.

The WHOQOL-BREF questionnaire was used to assess QoL parameters in five domains: global, physical, psychological, social and environmental. Perceived QoL was rated significantly worse by OBP patients compared to axSpA patients (*p* < 0.001; see Table 3). In the physical health domain, surveyed patients from both groups scored significantly lower compared to published values from the general population (27), with OBP patients again scoring significantly lower than axSpA patients [*p* < 0.0001, (OBP 50.05, axSpA 61.55, norm 73.5 points), see also Figure 1A]. OBP patients also reported a significantly worse mental-health related QoL than axSpA patients (OBP 61.62, axSpA 69.87, *p* < 0.001, norm 70.6 points). Also in the environment domain the OBP patients scored significantly lower than the axSpA patients (*p* < 0.001). Regarding social relationships, there were no significant differences between both groups (OBP 68.49, SpA 72.32, norm 71.5 points). Satisfaction with health was significantly worse in the OBP cohort than in the axSpA cohort (*p* < 0.001).

**Table 3:** Quality of life and depressive symptoms.

	Total (*n* = 300)	axSpA (*n* = 150)	OBP (*n* = 150)	value of *p*
Subjective QOL, mean (SD)	3.28 (0.90)	3.57 (0.84)	2.96 (0.86)	<0.001
Satisfaction with health, mean (SD)	2.73 (1.03)	3.07 (1.00)	2.37 (0.93)	<0.001
WHOQOL D1 (physical health), mean (SD)	56.0 (20.9)	61.55 (19.81)	50.05 (20.39)	<0.001
WHOQOL D2 (mental health), mean (SD)	66.0 (18.9)	69.87 (17.67)	61.62 (19.32)	<0.001
WHOQOL D3 (social), mean (SD)	70.5 (19.0)	72.32 (18.04)	68.49 (19.93)	0.099
WHOQOL D4 (environment), mean (SD)	73.6 (15.9)	76.81 (13.71)	69.96 (17.33)	<0.001
Depressive symptoms (Phq9), mean (SD)	7.52 (5.29)	6.71 (4.90)	8.50 (5.59)	0.006
Mental health (Ghq12), mean (SD)	11.3 (5.90)	10.07 (5.20)	12.83 (6.32)	<0.001

axSpA, axial spondyloarthritis; OBP, orthopedic back pain; QOL, quality of life; SD, standard deviation; WHOQOL, WHO quality of life questionnaire; D1, domain 1; VAS, visual analogue scale.

In the PhQ9, which assesses signs of depressive symptoms, the axSpA patients scored an average of 6.71 points, while the OBP patients reached on average 8.50 points and thus showed significantly more depressive symptoms (*p* = 0.006). Moderate to severe depressive symptoms could be detected in 42.9% of OBP, respectively 25% of axSpA patients (see Figure 1B). OBP patients reported significantly more difficulty to concentrate (*p* = 0.019), dejection, melancholy or hopelessness (*p* < 0.001) and little interest or pleasure in activities (*p* < 0.001).

Depressive symptoms showed a highly significant negative correlation with all four domains of QoL of the WHOQOL questionnaire (physical health, mental health, social relationships, environment) as well as with subjective QoL (Q1) and satisfaction with health (Q2; each *p* < 0.001). Functional limitation in everyday life (FFbH) also correlated significantly with all four domains (see correlation matrix, Figure 1C).

### 3.4. Predictors of quality of life

To determine factors predicting subjective QoL, a linear regression was conducted using stepwise backwards elimination, adjusting for sex, BMI and smoking status. The model explained 55.6% of variance in subjective QoL, adj. *R^2^* = 0.537, *F*(8, 189) = 29.60, *p* < 0.001. The results for individual predictors are shown in Table 4. Functional capacity in everyday life positively predicted subjective QoL, whereas depressive symptoms, diagnosis of OBP, pain level and age were inversely associated with subjective QoL.

**Table 4:** Multiple linear regression analysis of factors associated with subjective quality of life.

	Predictors	*β*	95% CI for *β*	*B*	SE	*p*
CBP (whole cohort)	Depressive symptoms (Phq9)	−0.33	−0.46 – −0.21	−0.05	0.01	<0.001
	Functional capacity (FFbH)	0.28	0.14–0.41	1.28	0.31	<0.001
	Diagnosis OBP	−0.25	−0.46 – −0.05	−0.22	0.09	0.016
	Pain (VAS 0–100)	−0.20	−0.32 – −0.09	−0.01	0.001	<0.001
	Age	−0.17	−0.28 – −0.06	−0.01	0.003	0.004
	*R*^2^ 0.556, adj. *R*^2^ = 0.537, *F*(8, 189) = 29.60, *p* < 0.001^1^					
axSpA	**Predictors**	***β***	**95% CI for *β***	***B***	**SE**	***p***
	BASDAI	−0.30	−0.49 – −0.11	−0.12	0.04	0.002
	Depressive symptoms (Phq9)	−0.25	−0.41 – −0.08	−0.04	0.01	0.004
	Age	−0.22	−0.37 – −0.08	−0.01	0.004	0.002
	Functional capacity (FFbH)	0.21	0.02–0.40	0.96	0.43	0.027
	*R*^2^ 0.532, adj. *R*^2^ 0.504, *F*(7, 117) = 18.99, *p* < 0.001^2^					
OBP	**Predictors**	***β***	**95% CI for *β***	***B***	**SE**	***p***
	Depressive symptoms (Phq9)	−0.39	−0.63 – −0.16	−0.06	0.02	0.001
	Functional capacity (FFbH)	0.28	0.04–0.52	1.23	0.54	0.025
	Pain (VAS 0–100)	−0.24	−0.44 – −0.03	−0.006	0.003	0.025
	*R*^2^ 0.502, adj. *R*^2^ 0.447, *F*(7, 64) = 9.21, *p* < 0.001^3^					

^1^Linear regression adjusted for sex, BMI, smoking status. ^2^Linear regression adjusted for sex, BMI, smoking status. ^3^Linear regression adjusted for sex, BMI, smoking status and age. CBP, chronic back pain; axSpA, axial spondyloarthritis; OBP, orthopedic back pain; VAS, visual analogue scale; *β*, standardized estimate; CI, confidence interval; B, estimate; SE, standard error.

In addition, separate multivariate models were calculated for the axSpA and OBP subgroups. For axSpA, disease activity assessed by BASDAI score, depressive symptoms, age and functional capacity were significant predictors of subjective QoL, whereas in OBP patients depressive symptoms, functional capacity and pain assessed on a visual analogue scale were significant predictors. The results for individual predictors are shown in the middle and bottom panel of Table 4.

### 3.5. Sex differences

**Figure 2:**
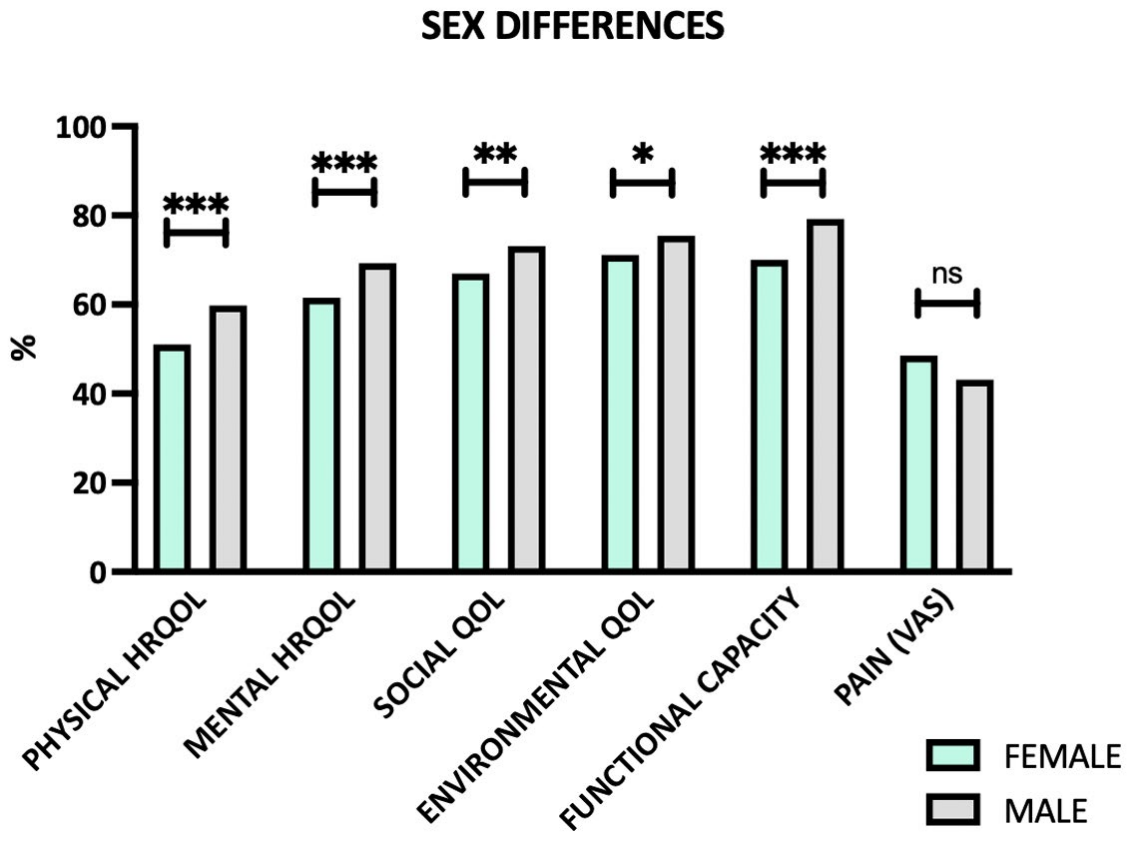
Sex differences regarding quality of life domains, functional capacity and pain. HRQOL, health-related quality of life; QOL, quality of life; VAS, visual analogue scale; ns, not significant. *p ≤ 0.05, **p ≤ 0.01, ***p ≤ 0.001.

Female patients were significantly less satisfied with their health compared to male patients (*p* = 0.001) and reported significantly worse QoL with respect to all four domains of QoL compared to men (*p* < 0.001, *p* < 0.001, *p* = 0.007, *p* = 0.025; Figure 2). In addition, women reported more functional limitations in daily life (*p* < 0.001) as well as significantly more depressive symptoms than male patients (*p* = 0.002). Reported pain intensity did not differ significantly between male and female patients (*p* = 0.150).

However, when stratifying the data by diagnosis, it could be shown that sex differences are in fact more pronounced in axSpA patients than in OBP patients (Table 5). Nonetheless, female OBP patients also reported significantly less satisfaction with health and more depressive symptoms (*p* = 0.030, respectively *p* = 0.004), while domains of QoL and functional capacity did not differ significantly compared to their male counterparts.

**Table 5:** Sex differences in quality of life, functional capacity and depression.

	Female (*n* = 128)		Male (*n* = 172)		value of *p*	
Diagnosis	axSpA (*n* = 61)	OBP (*n* = 67)	axSpA (*n* = 89)	OBP (*n* = 83)	axSpA	OBP
Subjective QOL, mean (SD)	3.47 (0.82)	2.85 (0.81)	3.65 (0.86)	3.05 (0.88)	0.207	0.174
Satisfaction with health, mean (SD)	2.84 (0.92)	2.18 (0.85)	3.24 (1.03)	2.53 (0.97)	**0.015**	**0.030**
WHOQOL D1 (physical health), mean (SD)	55.72 (18.95)	46.31 (17.95)	65.72 (19.45)	53.09 (21.82)	**0.003**	0.055
WHOQOL D2 (mental health), mean (SD)	64.63 (17.08)	58.42 (20.00)	73.63 (17.22)	64.21 (18.49)	**0.002**	0.087
WHOQOL D3 (social), mean (SD)	68.43 (19.68)	65.45 (20.08)	75.05 (16.37)	70.95 (19.59)	**0.030**	0.118
WHOQOL D4 (environment), mean (SD)	73.71 (14.05)	68.49 (17.51)	79.02 (13.10)	71.19 (17.21)	**0.022**	0.383
Functional capacity (FFbH), mean (SD)	76.2 (18.7)	64.1 (19.5)	84.0 (17.6)	74.1 (20.3)	**0.013**	0.150
Depressive symptoms (Phq9), mean (SD)	8.05 (4.79)	9.30 (5.61)	5.75 (4.78)	7.82 (5.52)	**0.005**	**0.004**
Pain VAS (0–100)	44.34 (27.29)	53.60 (32.04)	40.59 (26.62)	46.46 (33.13)	0.407	0.246

Bold values are statistically significant with a *p* < 0.05. axSpA, axial spondyloarthritis; OBP, orthopedic back pain; QOL, quality of life; SD, standard deviation; WHOQOL, WHO quality of life questionnaire; D1, domain 1; VAS, visual analogue scale.

## 4. Discussion

Chronic back pain constitutes a significant worldwide health burden. Healthcare costs as well as societal costs caused by lost productivity are substantial and growing (28, 29). The Global Burden of Disease 2010 Study identified low back pain as the leading cause of disability worldwide (30). Within this study, we compared the burden of disease, functional status, QoL and symptoms of depression in 150 patients with axSpA to a cohort of 150 orthopedic patients with chronic back pain and identified factors associated with QoL.

In both groups, the great majority of patients reported pain of mixed pain character, i.e., neither purely inflammatory nor purely mechanical symptoms of back pain. Furthermore, almost half of OBP patients reached 3 or more points in the inflammatory back pain questionnaire, suspicious of spondyloarthritis, despite having a clear orthopedic etiology of back pain. This mirrors the data of Riksman et al., who were unable to clearly discriminate between mechanical and inflammatory back pain by questionnaire in a chiropractic setting (7). This overlap in disease symptoms and the sometimes poor distinguishability between mechanical and inflammatory back pain likely contribute to the still frequent delay in diagnosis of inflammatory back pain and axSpA itself. Especially in older patients with concurrent mechanical causes of back pain, the diagnosis of axSpA can be difficult. Furthermore, in axSpA patients postinflammatory changes such as syndesmophytes and ankylosis may eventually lead to mechanical pain and axSpA may precipitate degenerative spinal changes including spinal stenosis and fractures (31). The correct identification of the origin of pain is essential as management differs considerably.

In this study, OBP patients were significantly older, had a significantly worse functional capacity in everyday life and a lower educational status than axSpA patients. This is in line with other studies reporting mechanical/orthopedic chronic back pain to be associated with age and inversely associated with educational degree (15, 32). In contrast, in axSpA 92% of patients have an onset of disease below 45 years of age and age of onset <45 years was included in the ASAS criteria for axSpA (33). Accordingly, the onset of symptoms is an important aspect in the differentiation of inflammatory from mechanical back pain.

Previous studies have shown that even patients with low-level but chronic back pain report an impaired QoL (34). In this study, physical health-related QoL was significantly reduced in both groups compared to published standard values (27), however even more so in the OBP group. A reduced health-related QoL has been previously reported, both for chronic mechanical back pain as well as for axSpA patients (35, 36). Furthermore, a recent similar study comparing non-specific low back pain (LBP) to axSpA patients also found LBP patients to have a reduced health-related QoL and more disability compared to axSpA patients (37). In contrast, Santos et al. reported higher levels of disability in axSpA compared to chronic LBP patients in a Portuguese community-based setting (38), whereas health-related QoL did not differ between axSpA and chronic LBP in the same cohort (39). In this study, OBP patients rated their subjective QoL as significantly worse than axSpA patients and were also less satisfied with their health. In a multivariate linear regression model, predictors of subjective QoL included depressive symptoms, functional capacity, orthopedic back pain, pain intensity and age. Separate models calculated for axSpA and OBP in fact identified largely the same predictors for both groups, although age did not contribute significantly to subjective QoL in the OBP subgroup.

Pain and disability were also identified as prominent predictors of lower QoL in other studies on chronic back pain (16). In axSpA, a number of studies identified functional capacity (BASFI) and disease activity (BASDAI or ASDAS) as the main predictors of QoL (35, 40). Data on age are inconsistent, with some studies reporting an association of younger age with higher QoL in back pain patients, while others described older age to be associated with better well-being (well-being paradox) (36, 41). These inconsistencies can be explained by the heterogeneity of the patient cohorts analysed. For axSpA, a significant association of QoL with age has not been described and disease duration has explicitly been reported not to be significantly associated with QoL (35).

While in our multivariate regression model sex was not an independently associated factor, it has furthermore been shown for both, chronic mechanical back pain and axSpA, that female patients report a reduced QoL compared to male patients (35, 41). Also in our study, female patients reported a worse QoL regarding physical and mental health-related QoL as well as social and environmental QoL. Furthermore, female axSpA patients reported a significantly worse functional capacity in everyday life. Other studies in axSpA have, however, shown men to have more structural damage (42). Interestingly, Lopez-Medina et al. (35) identified structural damage (assessed by mSASSS) to be associated with a better QoL (35). They hypothesized that patients with more damage and disability may have adjusted their everyday lives accordingly (habituation) and may thus also perceive less pain. Furthermore, syndesmophytes opposed to erosions have been hypothesized to cause less pain. However, men were also shown to be diagnosed earlier and have a better therapy response to anti-TNF treatment (14), which may contribute to the observed differences in functional capacity and QoL.

Proinflammatory cytokines and chronic inflammation have been implicated in the development of pain as well as depression. Nonetheless, in this study axSpA patients showed fewer depressive symptoms than OBP patients. However, independently of back pain character, depressive symptoms were common in this cohort. Similarly, a Canadian study reported a rate of major depression of 19.8% for chronic back pain patients (43). In other studies, it was reported that up to 45% of back pain patients met the criteria for depression (44). Likewise, for axSpA data on prevalence of depression are heterogeneous, depending on the employed criteria and thresholds (45). AxSpA patients with depression have been shown to have higher disease activity and more functional impairment (45). Furthermore, higher levels of depression have been reported to be associated with higher perceived pain intensity and disability (18). In this study, depressive symptoms were a significant negative predictor of subjective QoL. However, also other psychological factors seem to play a role regarding QoL in chronic back pain patients. Agnus Tom et al. described an individual’s beliefs regarding the causes and outcome of pain to have a significant impact on QoL (17). A number of studies furthermore identified kinesiophobia/fear avoidance belief, i.e., avoidance of physical activity due to fear of pain as another psychological factor, which is inversely associated with QoL and correlated with functional impairment or disability (46). In contrast, physical activity in leisure time was shown to have a positive impact on QoL for chronic mechanical back pain and also for axSpA patients (47). Thus, proper patient education and physiotherapy or exercise programs should be incorporated into the treatment of back pain irrespective of cause. These measures may help to decrease pain, reduce psychological comorbidity and prevent functional impairment and disability.

Limitations of this study include the monocentric study design, which led to a comparatively small patient number, as well as the questionnaire-based approach. Furthermore, it needs to be taken into account that the great majority of axSpA patients were under treatment, whereas the orthopedic cohort was a mixed cohort of treated and untreated patients at the time of survey owing to the study design. The cross-sectional approach does not allow to capture potential improvement over time with recommended treatments especially in the orthopedic cohort with patients undergoing surgery or injection therapy. At this point we also want to underline again that with increasing age and disease duration axSpA patients may also have mechanical or unspecific back pain. Furthermore, the university hospital setting may have led to a bias and data might not be directly transferable to other outpatient settings or GP practices.

In conclusion, in this study chronic back pain was associated with a high morbidity, reduced QoL and increased depressive symptoms regardless of the pain character, with significant differences between male and female patients. Awareness and addressing of the predisposing factors identified in this study may help to overcome unmet needs and improve QoL for these patients.

## Data availability statement

The raw data supporting the conclusions of this article will be made available by the authors, without undue reservation.

## Ethics statement

This study was conducted under the ethics protocol 37/17n of the ethics committee of the University of Freiburg, Germany. Patients gave their written informed consent to participate according to International Conference on Harmonization Good Clinical Practice (ICH GCP) guidelines.

## Author contributions

NV, RV, JT, SF, GH, DE, and LE designed and supervised the study and gave critical input. SH, SF, NV, NF, NC, MS, IR, and JT recruited, consented and cared for the patients enrolled in this study and provided clinical information. NF, SH, and NV analysed the data. NF and NV wrote the manuscript. All authors read and approved the final manuscript.

## Funding

Parts of this study were financially supported by an unrestricted grant from Novartis Pharma GmbH, Germany. The funder was not involved in the study design, collection, analysis, interpretation of data, the writing of this article or the decision to submit it for publication.

## Conflict of interest

NV: Speaker honoraria: AbbVie, Novartis, UCB, Bristol-Myers-Squibb, Pfizer; Advisory boards: AbbVie, Novartis, UCB; Research grants: Bristol-Myers-Squibb, Novartis, Pfizer. JT: Speaker honoraria: GSK, BMS, Astra-Zeneca, Abbvie, UCB, Lilly; Advisory boards: Novartis, GSK, Astra-Zeneca, Lilly. Grant/research support from: BMS, Novartis. RV: Speaker fees: AbbVie, Amgen, BMS, Boehringer-Ingelheim, GSK, Janssen-Cilag, Hexal, Novartis, Pfizer, Roche; Advisory boards: AbbVie, Amgen, Boehringer-Ingelheim, BMS, GSK, Janssen-Cilag, Hexal, Neutrolis, Novartis, Sanofi, Takeda; Unrestricted research grants: Amgen, BMS, Novartis, Pfizer. NF received travel grants from AbbVie, Janssen, Sobi, Pfizer.

The remaining authors declare that the research was conducted in the absence of any commercial or financial relationships that could be construed as a potential conflict of interest.

## Publisher’s note

All claims expressed in this article are solely those of the authors and do not necessarily represent those of their affiliated organizations, or those of the publisher, the editors and the reviewers. Any product that may be evaluated in this article, or claim that may be made by its manufacturer, is not guaranteed or endorsed by the publisher.

## Footnotes

^1^www.graphpad.com

^2^https://www.jamovi.org
